# Identification of an autophagy-related gene signature predicting overall survival for hepatocellular carcinoma

**DOI:** 10.1042/BSR20203231

**Published:** 2021-01-15

**Authors:** Wenfang Xu, Wenke Guo, Ping Lu, Duan Ma, Lei Liu, Fudong Yu

**Affiliations:** 1 Department of Biochemistry and Molecular Biology, School of Basic Medical Sciences and Institutes of Biomedical Sciences, Fudan University, Shanghai, China; 2 NHC Key Laboratory of Reproduction Regulation (Shanghai Institute of Planned Parenthood Research), Fudan University, Shanghai, China

**Keywords:** autophagy, biomarkers, hepatocellular carcinoma, prognosis, TCGA

## Abstract

The poor prognosis of hepatocellular carcinoma (HCC) calls for the development of accurate prognostic models. The growing number of studies indicating a correlation between autophagy activity and HCC indicates there is a commitment to finding solutions for the prognosis of HCC from the perspective of autophagy. We used a cohort in The Cancer Genome Atlas (TCGA) to evaluate the expression of autophagy-related genes in 371 HCC samples using univariate Cox and lasso Cox regression analysis, and the prognostic features were identified. A prognostic model was established by combining the expression of selected genes with the multivariate Cox regression coefficient of each gene. Eight autophagy-related genes were selected as prognostic features of HCC. We established the HCC prognostic risk model in TCGA dataset using these identified prognostic genes. The model’s stability was confirmed in two independent verification sets (GSE14520 and GSE36376). The model had a good predictive power for the overall survival (OS) of HCC (hazard ratio = 2.32, 95% confidence interval = 1.76–3.05, *P*<0.001). Moreover, the risk score computed by the model did not depend on other clinical parameters. Finally, the applicability of the model was demonstrated through a nomogram (C-index = 0.701). In the present study, we established an autophagy-related risk model having a high prediction accuracy for OS in HCC. Our findings will contribute to the definition of prognosis and establishment of personalized therapy for HCC patients.

## Introduction

Liver cancer is the sixth most common malignant tumor and the associated mortality rate was ranked fourth among cancer-related deaths in 2018 [1] and these rates have remained high for nearly 2 years [2]. Hepatocellular carcinoma (HCC) patients account for 75–85% of primary liver cancer. The 5-year average survival rate of HCC is below 18% [2]. Closely influenced by a variety of risk factors (such as hepatitis, smoking, and alcohol), the prognosis of HCC patients is highly variable [3–8]. Due to the complex pathogenic factors of HCC, accurate prognosis is difficult [9,10]; therefore, establishing a useful prognostic model is an urgent need [11].

In the European Association for the Study of the Liver (EASL) guidelines, AFP, VEGF, and Angiopoietin-2 have been suggested as prognostic markers for HCC. Keratin-19 and EpCAM have been suggested as candidate prognostic biomarkers because of their correlation with the poor prognosis of HCC patients [12]. However, even the most common marker, alpha-fetoprotein (AFP), was only abnormally expressed in 70% of HCC patients [13]. The prediction accuracy of HCC prognostic markers still needs to be improved. In recent years, studies on multi-gene prognostic markers for HCC have been reported. For example, Liu et al. screened a four-gene metabolic signature [14]. Although these multi-gene combinations have not been used to guide clinical practice, the studies remind researchers that multiple-gene combinations have the potential to become more accurate prognostic markers for HCC.

Autophagy is a mechanism used by eukaryotic cells to maintain homeostasis and is a process of self-degradation that occurs when cells are damaged, undernourished or defective [15]. Abnormalities in autophagy have been associated with the molecular pathogenesis of cancer occurrence and development [16]. In recent years, preclinical studies have provided evidence on the role of autophagy related processes in HCC prognosis [17–19]. Among them, studies on autophagy inhibitors as therapeutic agents in HCC models have shown promising results [20–22]. Cumulative evidence indicates there is a commitment to finding solutions for the prognosis of HCC from the perspective of autophagy.

In the present study, we used the mRNA data and clinical information of HCC from The Cancer Genome Atlas (TCGA) and Gene Expression Omnibus (GEO) datasets to develop a prognostic risk model from the perspective of autophagy. The effectiveness of the prognostic model was evaluated, and then the independence, robustness and reliability of this model were demonstrated. Finally, a nomogram was established to facilitate potential clinical applications.

## Materials and methods

### Data collection and preprocessing

The level-3 mRNA expression data and corresponding phenotype data of 371 primary HCC samples in TCGA cohort were downloaded from the University of California Santa Cruz (UCSC) Xena database (https://tcga.xenahubs.net/download/TCGA.LIHC.sampleMap). Of these, 365 samples included prognostic information. The gene expression value was transformed by log2 (normalized RSEM count + 1). Then, the genes with low or no expression were removed from the analysis (that is, genes with an average count value greater than 1 and expressed in more than 75% patients were included). The standardized mRNA data of GSE14520 and GSE36376 were acquired from the GEO database (https://www.ncbi.nlm.nih.gov/geo/). Specifically, these datasets comprised 221 HCC samples processed on the Affymetrix HT Human Genome U133A microarray platform in GSE14520, and 223 HCC samples processed on the HumanHT-12 v4 Expression BeadChips array platform in GSE36376, which were used as validation sets. Quality controls included Relative Log Expression (RLE) and Normalized Unscaled Standard Error (NUSE) implemented in the affyPLM package available from Bioconductor (www.bioconductor.org). Raw gene expression data were background corrected using the Robust Multi-Array Average (RMA) method, standardized by the Quantiles method, and summarized by the median polish method. The above methods were all implemented in the affy package available from Bioconductor. According to the annotation files in the platform provided by the chip manufacturer (GPL3921 for GSE14520, GPL10558 for GSE36376), the probe labels were converted into gene symbols. The average expression values were set as relative expression values of genes which were matched with multiple probes. In addition, 232 and 328 autophagy-related genes were obtained from the Human Autophagy Database (HADb) and the gene set GO_regulation_of_autophagy (M10281) in the Molecular Signatures Database v7.1 (MSigDB), respectively. Overlapping genes in the two gene sets were removed, and thus 494 autophagy-related genes remained for the analysis.

### Screening characteristic genes and modeling

Based on TNM staging, stage I and stage II patients from the 371 primary HCC samples in TCGA were combined as the control group. To identify the differentially expressed genes in samples of advanced patients (stage III and stage IV), Bayes test (using an FDR<0.05 cutoff) was conducted. The autophagy genes related to HCC prognosis were initially obtained by intersecting the above differentially expressed genes and the 494 autophagy genes. Univariate regression analysis (*P*<0.05) was performed to identify candidate autophagy-related prognostic genes related to OS. Lasso Cox regression was used to confirm the final prognostic signature [23]. Genes identified in the univariate analysis as covariates were included in the multivariate Cox regression analysis to determine their impact on the OS. The prognostic model was established by combining the expression values and the multivariate Cox regression coefficients of the selected genes, that is, the risk score for each patient is equal to the sum of the gene expression values multiplied by the regression coefficients.

### Evaluation of the model prediction effect

The median risk score was used as the threshold to stratify HCC patients. Patients with a risk score greater than the threshold value were assigned to the high-risk group, and the remaining patients were assigned to the low-risk group. The difference in survival between the two groups was evaluated by a log-rank test. Survival curves were drawn using the Kaplan–Meier (K-M) method. The time-dependent receiver operating characteristic (ROC) curve was used to evaluate the specificity and sensitivity of the risk score in predicting the survival rate of HCC at the 1-, 3-, and 5-year follow-up. At the same time, the respective effects of risk score and other clinical indicators (age, sex, AFP, TNM stage, histological grade, and vascular tumor infiltration) were assessed to predict the one-year survival rate of HCC. The ability of the model to predict OS was verified in the validation datasets (GSE14520 and GSE36376). In addition, based on the increase in the risk value, the distribution of patient death events was displayed using a dot plot. A heatmap was used to view the expression distribution of each characteristic gene in the two different risk groups.

### Detecting the independence and reliability of the prognostic model

We selected clinical indicators (age, sex, AFP, TNM stage, histological grade and vascular tumor infiltration) commonly used in the prognosis of HCC. Among these possible prognostic factors, the risk score (14.0–17.5) and age (range, 16–90 years) were used as continuous variables, and sex (male/female), AFP (≥400/<400 ng/mL), TNM stage (III+IV/I+II), histologic grade (4/3/2/1), and vascular tumor invasion (Macro/Micro/None) were transformed into categorical variables. Univariate and multivariate Cox regression methods for clinical properties and risk score of HCC patients in the TCGA cohort were performed. We identified clinical factors related to survival. The Log-rank test was used to verify whether the risk score was related to other survival-related clinical information. Then, variables that could be used as independent prognostic factors were used to build a nomogram. Subsequently, the prediction accuracy of the 1-, 3-, and 5-year survival rate was calculated by comparing the consistency of the predicted value and its true value. Based on the differentially expressed genes between the high- and low-risk groups, we performed KEGG pathway enrichment analysis using the Gene Set Enrichment Analysis (GSEA).

### Statistical analysis

All statistical analysis methods were performed using R, version 3.6.1. The processing of microarray sequencing data depended on the R package ‘GEOquery’. The package ‘edgeR’ was used for differential gene screening. Univariate and multivariate Cox regressions were analyzed by the ‘survival::coxph’ function. Lasso-Cox regression was analyzed by R package ‘glmnet’. In addition, the log-rank test was carried out using the survdiff function in the ‘survival’ package. And time-dependent ROC was analyzed by the ‘timeROC’ package. The characteristic genes expression heatmap was plotted by the ‘ggplot::heatmap’ function. The establishment and application of the nomogram were achieved using the R package ‘rms’. The GSEA used for pathway enrichment was performed with the R package ‘clusterProfiler’.

## Results

### Autophagy-related prognostic gene screening

**Figure 1:**
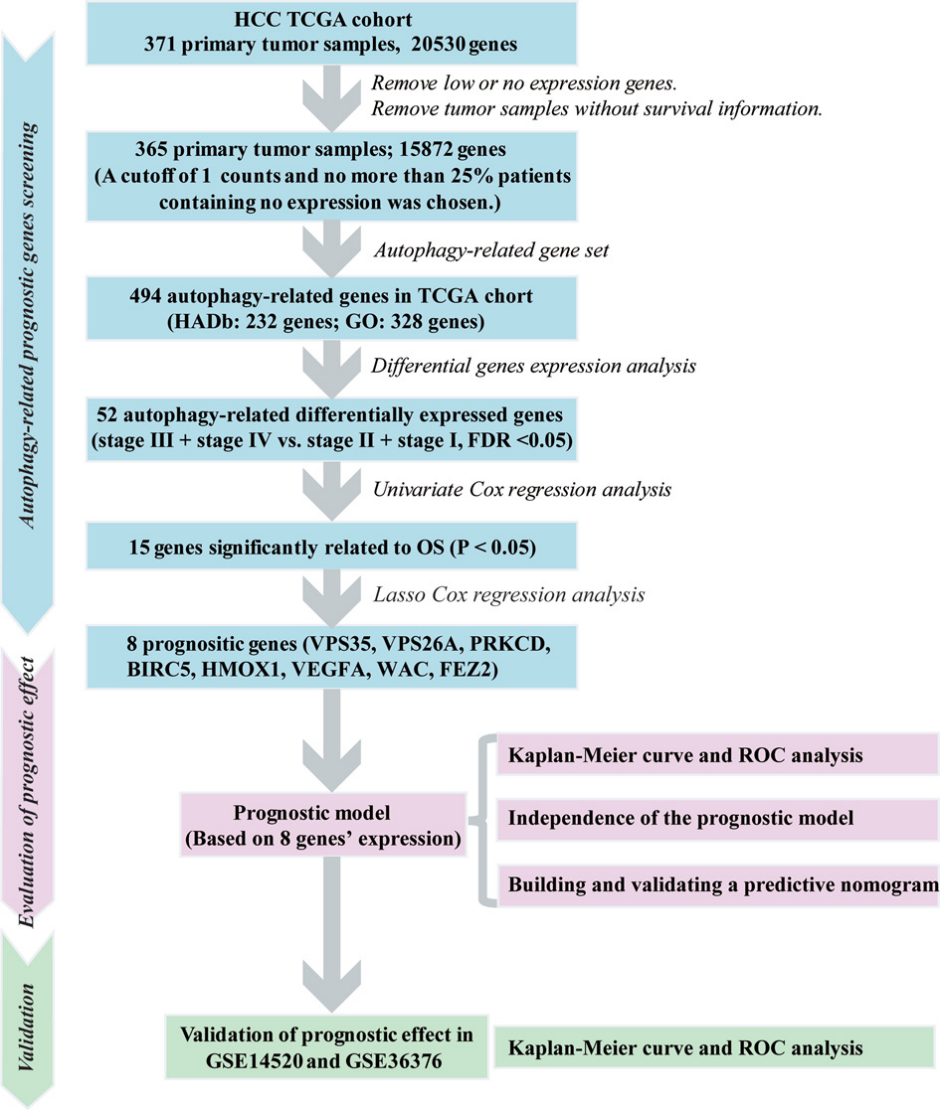
Flow chart showing screening of the autophagy-related gene signature to predict survival of HCC patients

**Figure 2:**
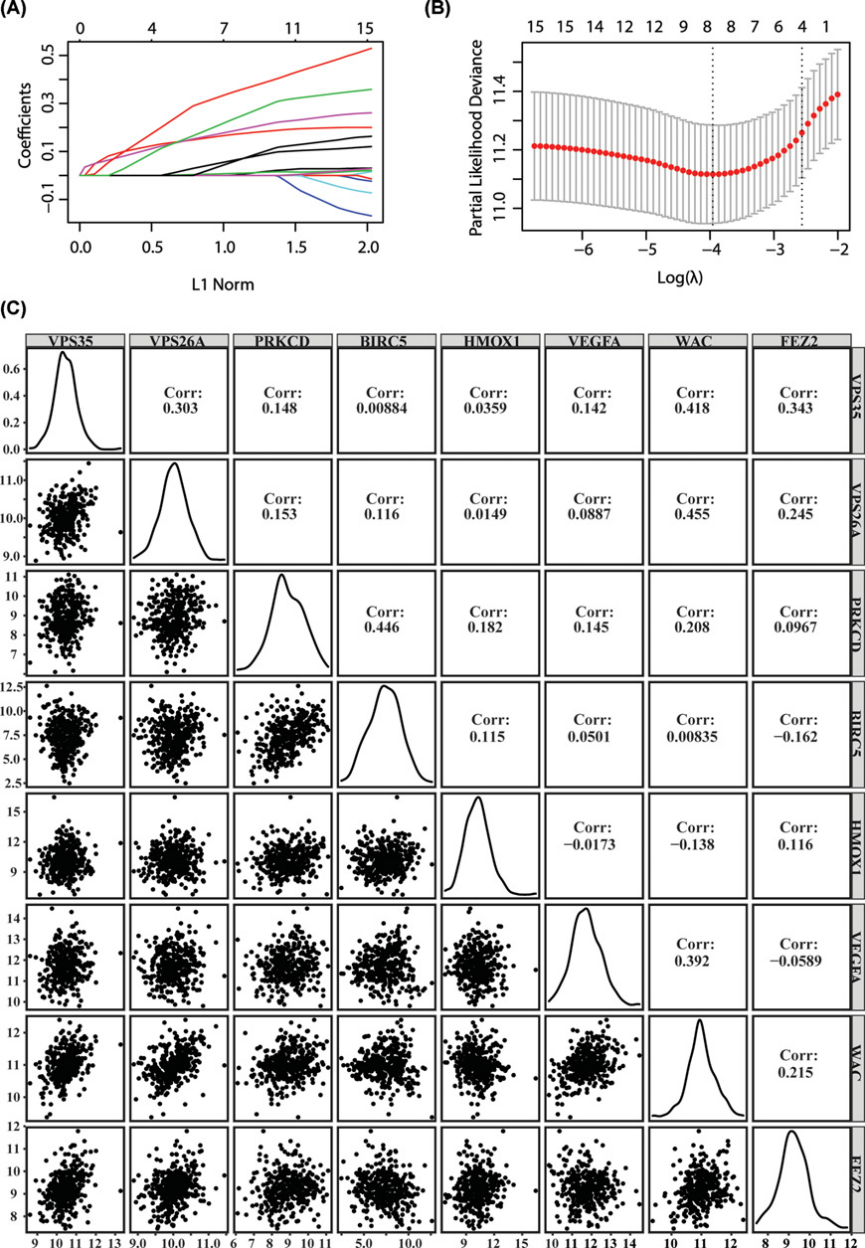
Lasso-Cox regression analysis was used to screen the prognostic genes In Lasso-Cox regression analysis, for the regression coefficients of each gene, positive numbers are positively correlated, negative numbers are negatively correlated (**A**). Selecting the best parameter (λ). (**B**) In Lasso: performing Pearson correlation coefficient analysis between gene expression values (**C**).

We analyzed the transcriptome data of 371 primary HCC samples in TCGA to identify prognostic genes. To identify autophagy genes related to the OS of HCC, the differentially expressed genes (FDR<0.05) between advanced stage (III + IV) and early stage patients (I + II) were analyzed, and 52 genes were obtained after the intersection with the 494 autophagy-related genes. Next, univariate Cox regression analysis was used, and 15 autophagy genes related to OS were obtained (*P*<0.05, Table 1). These candidate prognostic genes were screened by Lasso Cox regression analysis, and finally eight prognostic genes (including VPS35, VPS26A, PRKCD, BIRC5, HMOX1, VEGFA, WAC and FEZ2) were obtained (Figures 1 and 2A,B).

**Table 1:** Fifteen autophagy-related candidate HCC prognosis genes

Gene symbol	HR (95% CI)*	*P*-value
VPS35	1.59 (1.16–2.18)	0.004
VPS26A	2.33 (1.48–3.66)	<0.001
PRKCD	1.42 (1.17–1.72)	<0.001
HIF1A	1.19 (1.01–1.39)	0.035
ERO1L	1.22 (1.00–1.49)	0.048
BIRC5	1.22 (1.11–1.35)	<0.001
HMOX1	1.15 (1.01–1.31)	0.033
SNX6	1.42 (1.01–1.99)	0.045
HK2	1.11 (1.03–1.20)	0.008
DNM1L	1.47 (1.04–2.07)	0.028
BAK1	1.31 (1.09–1.58)	0.005
FBXL2	1.11 (1.01–1.23)	0.038
VEGFA	1.29 (1.02–1.62)	0.032
WAC	2.01 (1.34–3.00)	0.001
FEZ2	1.39 (1.10–1.75)	0.006

* CI, confidence interval; HCC, hepatocellular carcinoma; HR, hazard ratio.

**Figure 3:**
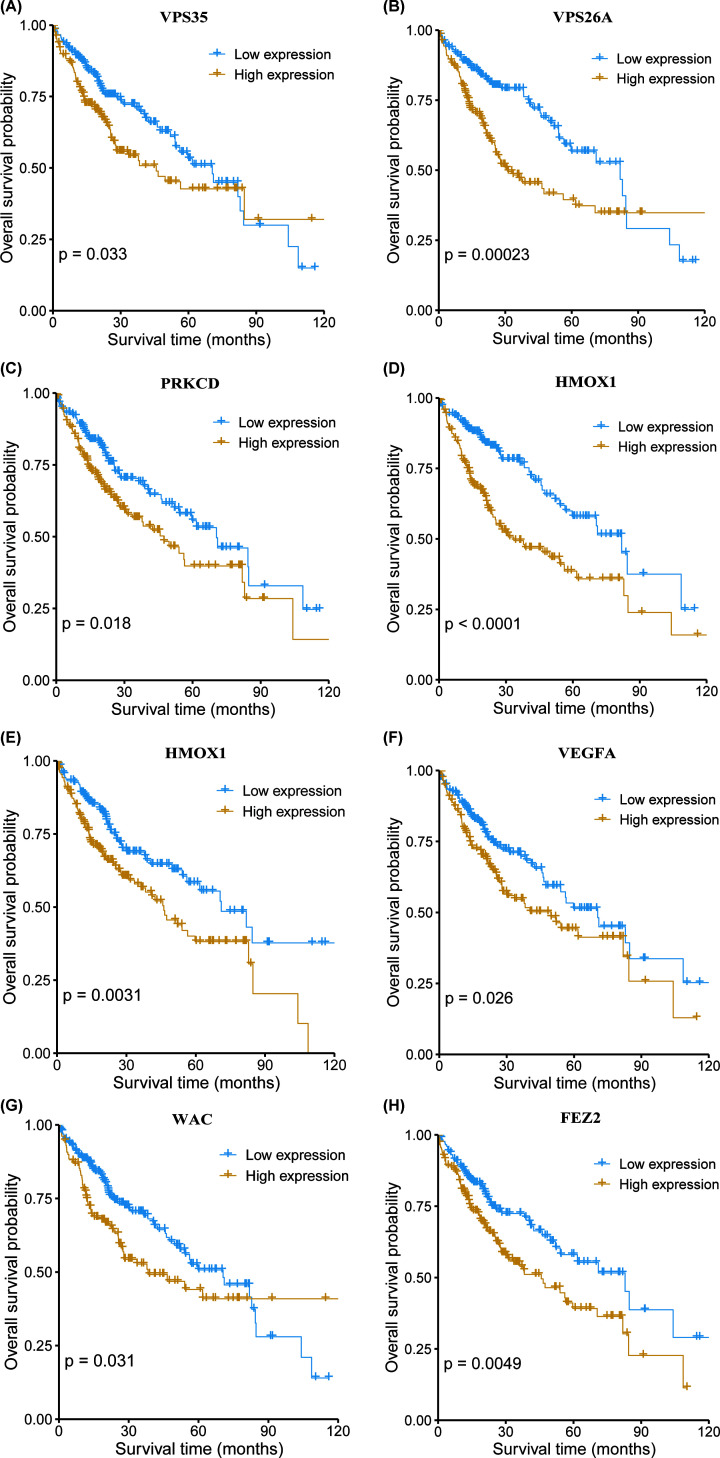
The correlation between each of the eight autophagy-related prognostic genes and the prognosis of HCC Kaplan–Meier analysis of patient survival comparing the high expression and low expression groups of each prognostic gene (**A–H**).

Furthermore, we performed a Pearson correlation analysis on prognostic genes’ expression and found that the genes are independent of each other, indicating that there is no redundancy between these prognostic genes (Figure 2C, correlation<0.5). To define the role of each gene in the prognosis of HCC, we performed a differential analysis of patient survival comparing the high expression and low expression groups of each prognostic gene. The results showed that the eight identified prognostic genes were all adverse factors for HCC survival (Figure 3).

### Establishment and evaluation of the prognostic model

**Figure 4:**
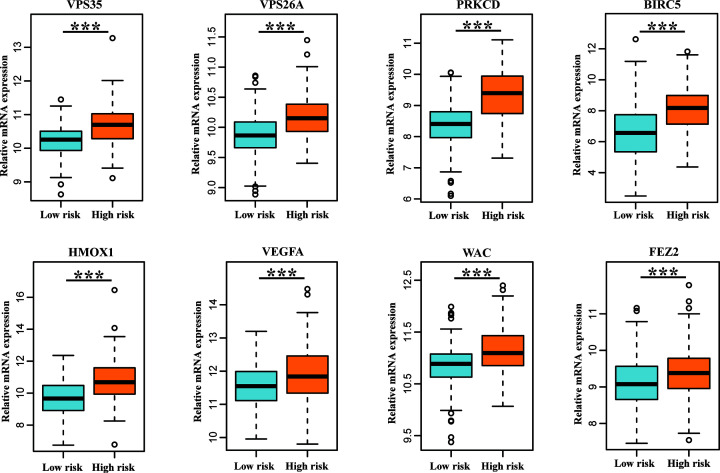
Differential expression analysis of the identified prognostic genes between the high-risk group and the low-risk group The results showed that all eight genes were up-regulated in the high-risk patient group. *** means significant difference, *P*<0.001.

**Figure 5:**
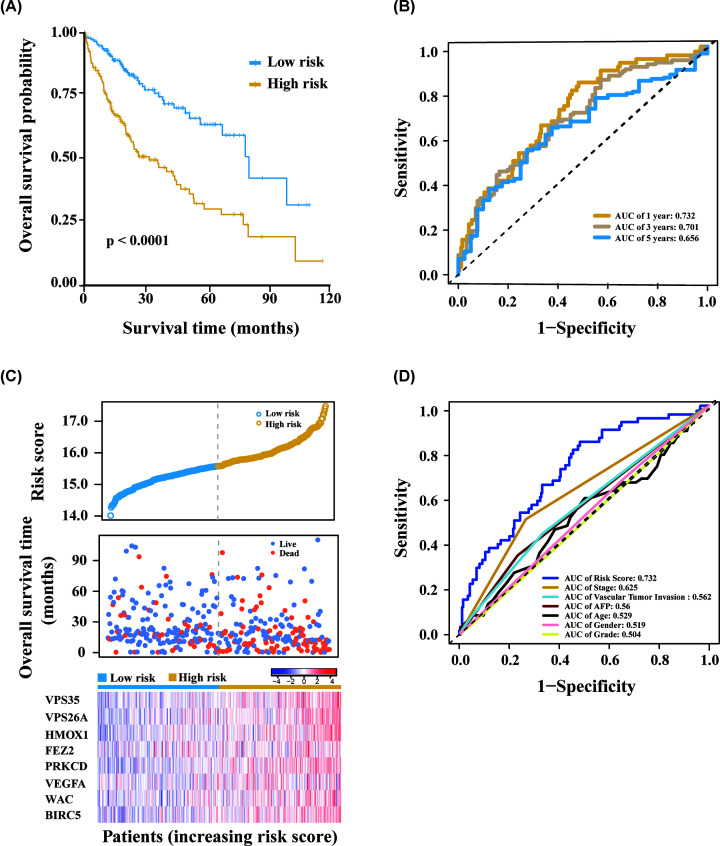
Autophagy-related prognosis genes are significantly correlated with the overall survival of HCC Kaplan–Meier analysis between high- and low-risk groups of TCGA HCC patients, the overall prognosis of HCC patients with high risk score is poor (**A**). ROC analysis of the risk score to assess the sensitivity and specificity (**B**). The relationship between risk score, death, and expression of characteristic genes (**C**). The AUC values of risk score and clinical indicators at the one-year OS are displayed (**D**).

We then constructed an autophagy-related prognostic model based on the eight HCC prognostic signature genes (VPS35, VPS26A, PRKCD, BIRC5, HMOX1, VEGFA, WAC and FEZ2). Multivariate Cox regression analysis was performed including each prognostic gene, and the regression coefficient of each gene was obtained. The model was defined as follows: Risk Score = 0.0326*expression(VPS35) + 0.1966*expression(VPS26A) + 0.0213*expression(PRKCD) + 0.2453*expression(BIRC5) + 0.1205*expression(HMOX1) +0.1554*expression(VEGFA) + 0.4405*expression(WAC) + 0.3583*expression(FEZ2). Using TCGA cohort, 365 HCC samples were evaluated and each sample was given a risk score and assigned to a risk group. First, we performed a differential expression analysis of the identified prognostic genes between the high-risk group and the low-risk group. The results showed that all eight genes were up-regulated in the high-risk patient group (Figure 4). To assess the effects of the model, we conducted a survival differential analysis between the high- and low-risk groups. The results indicated that the prognosis of patients in the high-risk group was significantly worse than that in the low-risk group (*P*<0.0001; Figure 5A). In detail, the median OS of patients in the high-risk group was 17.3 months, while in the low-risk group the OS was 21.4 months. To further estimate the predictive performance of this risk model, time-dependent ROC analysis was performed for 1-, 3-, and 5- year OS. Their corresponding area under curve (AUC) values were 0.732, 0.701, and 0.656, respectively, which demonstrated the good performance of our model (AUC>0.5; Figure 5B). In our model, the higher the risk score, the earlier the patient's event occurs, and the higher the expression of the eight genes (Figure 5C). To compare the prognostic effects of the risk score with other clinical factors, time-dependent ROC analysis was performed for the 1-year OS. The results showed that the risk score had the best prognostic effect at this time point, with an AUC value of 0.732 (Figure 5D). The above results indicated that we had established an effective autophagy-related HCC prognosis model.

### Verification of the effect of prognostic model

**Figure 6:**
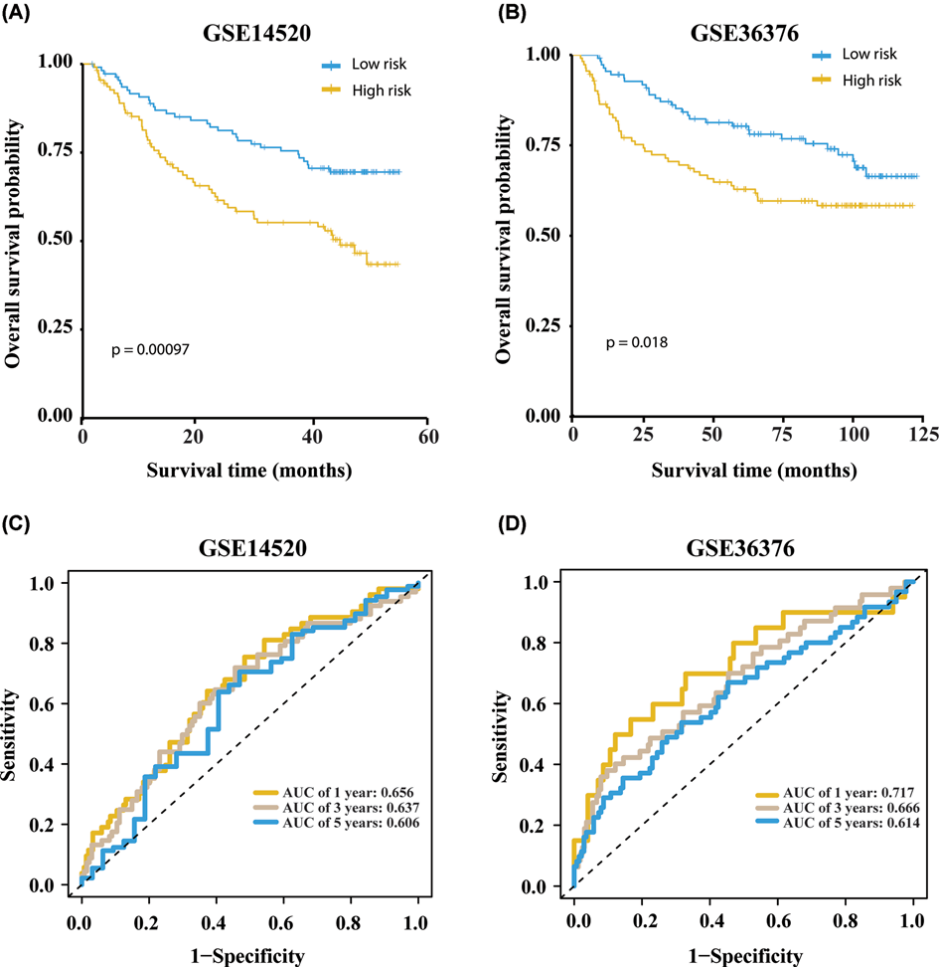
Verification of the validity of the prognostic model for overall survival The prognostic effect of the model was verified in the verification datasets GSE14520 (**A,C**) and GSE36376 (**B,D**).

To rule out the potential for overfitting of the model in TCGA, we verified the model in two independent data sets (GSE14520 and GSE36376). The median OS of patients in the high-risk group (36.5 months in GSE14520, 66.9 months in GSE36376) was significantly (*P*=0.00097 and *P*=0.018, respectively) shorter than the median OS value in the low-risk group (53.7 months in GSE14520, 84.1 months in GSE36376) (Figure 6A,B), which was consist with the results in the training set. In GSE14520 and GSE36376, the AUC values for 1-, 3-, and 5-year OS were 0.656, 0.637, 0.606, and 0.717, 0.666, 0.614, respectively (Figure 6C,D). The above results indicated that the autophagy-related risk model was robust across platforms.

### Verification of prognostic independence of the model

**Figure 7:**
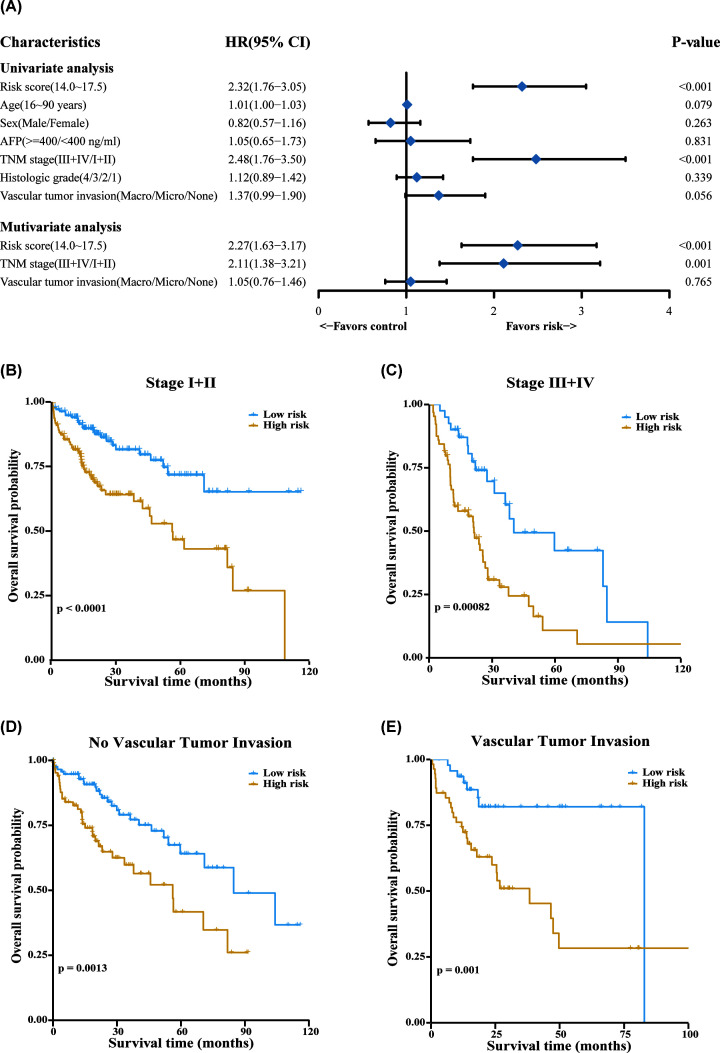
The prognostic risk score is an independent factor for overall survival in HCC Forrest plot of univariate and multivariate Cox regression analysis of HCC overall survival with various clinical indicators and risk score (**A**). Patients were classified by whether they presented vascular invasion and their TNM stage. Next, the performance of the risk score was evaluated for each subcategory (**B**–**E**).

We investigated whether the clinical characteristics and risk score of HCC patients in the TCGA cohort were related to prognosis. Univariate Cox regression analysis was conducted, the results indicated that risk score and TNM stage were significantly (*P*<0.001) correlated with OS. In addition, the correlation between vascular tumor invasion and OS showed a trend for significance (*P*=0.056). Finally, these three factors were used as covariates for the multivariate cox regression analysis, which showed that that the TNM stage (HR = 2.11, 95% CI = 1.38–3.21, *P*=0.001) and the risk score (HR = 2.27, 95% CI = 1.63–3.17, *P*<0.001) were independent prognostic factors for OS of HCC patients (Figure 7A). In addition, the risk score was an independent prognostic factor regardless of tumor stage or vascular invasion (Figure 7B–E), which also further illustrated the independent prognostic value of the risk model.

### Establishment and evaluation of nomogram

**Figure 8:**
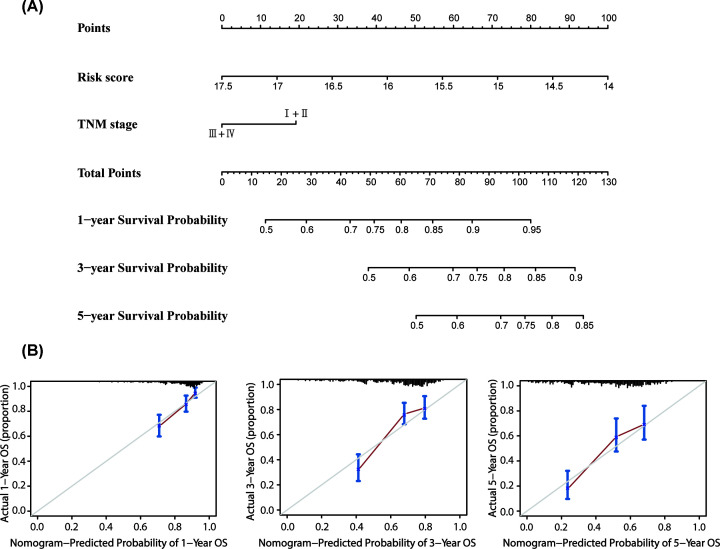
The nomogram constructed to predict overall survival (OS) in the clinical setting presents good prediction ability A nomogram created by the combination of the risk score and TNM stage to predict the OS of HCC (**A**). Calibration charts predicting 1-, 3- and 5-year survival in the training set. The horizontal axis and vertical axis represent the predicted survival probability and the actual survival probability (**B**).

To evaluate whether our model could effectively predict the prognosis of HCC patients in the clinical setting, we selected factors related to OS (risk score and TNM stage) of HCC and established a nomogram (Figure 8A) which could predict 1-, 3-, and 5-year survival. The C-index value of this nomogram model was 0.701. In addition, the calibration plots for 1-, 3-, and 5-year survival predictions showed that the nomogram model had satisfactory predictive performance (Figure 8B). Therefore, the nomogram model once again confirmed the reliability and prospective clinical applicability of the risk model.

### Molecular mechanism of autophagy-related risk model

**Figure 9:**
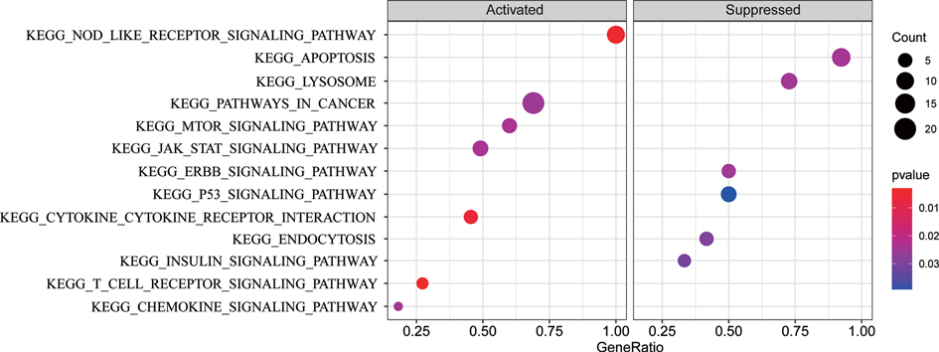
Gene Set Enrichment Analysis (GSEA) of the differentially expressed autophagy genes of high-risk patients in the KEGG pathways and modules

To explore the molecular mechanisms involved in the autophagy-related prognostic risk model, we compared the differentially expressed autophagy genes of high-risk patients with those of low-risk patients, and performed enrichment analysis in the KEGG pathway and module. The results (Figure 9) showed that the mTOR signaling pathway was up-regulated and lysosomal signaling was down-regulated in high-risk patients, indicating that autophagy activity may be inhibited in patients with poor prognosis and suggesting that autophagy mechanisms have a certain protective effect on HCC survival. In addition, our analysis of the differential expression of the classic autophagy markers ULK-1, Beclin-1, and LC3B in the high- and low-risk groups showed that the expression of ULK-1 was significantly down-regulated in the high-risk group (Table 2), further confirming that autophagy is suppressed in HCC patients with poor prognosis.

**Table 2:** The changes of classic autophagy markers ULK-1, Beclin-1, and LC3B in the high-risk group with poor prognosis of HCC

Gene Symbol	LogFC	LogCPM	P Value	FDR
ULK1	-0.169	6.228	0.0104	0.019*
BECN1	0.051	5.942	0.303	0.378
LC3B	0.125	5.860	0.084	0.123

* There is a significant difference; HCC, hepatocellular carcinoma.

In addition, several cancer-related pathways and modules were enriched (*P*<0.05) including the down-regulation of the cancer inhibitory P53 signaling pathway and the inhibition of apoptosis, as well as the up-regulation of the JAK-STAT signaling pathway, and the pathways in cancer. These results can provide an explanation for the poor survival of patients observed in the high-risk group at the molecular level, indicating that the risk model we established is reliable.

## Discussion

In recent years, studies have increasingly indicated that autophagy was involved in the occurrence and progression of HCC [17,18,24], but there has been no research on the role of multiple autophagy-related genes in HCC prognosis. We carried out this study taking advantage of the available high-throughput technology and multi-genes prediction methods for HCC survival [25–27]. According to the relative transcription levels of eight autophagy-related genes (VPS35, VPS26A, PRKCD, BIRC5, HMOX1, VEGFA, WAC, FEZ2) in TCGA cohort, we established an HCC prognostic risk model. This model was an independent predictor of OS (HR = 2.32, 95% CI = 1.76–3.05, *P*<0.001) compared with other prognostic indicators and effectively predicted the outcome of HCC. The stability of the model was confirmed in two independent verification datasets (GSE14520 and GSE36376), and its reliability was explained by the results of risk score-related genes enrichment analysis involving multiple cancer-related pathways. Finally, the clinical applicability of the model was also demonstrated through the construction of a nomogram. Therefore, our study provided a direction for the study of autophagy-related genes in HCC prognosis.

Among the eight autophagy-related prognostic genes we identified, VPS35 has been proposed as a potential new oncogene of HCC, as it promotes the liver tumor cell proliferation via PI3K/AKT signaling [28]. A previous study indicated that VPS26A might be associated with cancer prognosis [29]. The protein encoded by PRKCD was reported to be activated by diacylglycerol and acted as both a tumor suppressor and a positive regulator of cell cycle progression [30,31]. In a study on the anticancer properties of FZD7 after pharmacological inhibition of HCC, it was suggested that the mechanism may be associated with PRKCD mutations [32]. BIRC5 has been described as a negative regulator of apoptosis, and its expression was reported to be higher in most tumors including HCC [33–36], which is consistent with our findings indicating that the expression of BIRC5 is higher in the high-risk group of HCC prognosis. Moreover, the potential influence of BIRC5, HMOX1, and VEGFA in the prognosis of HCC had been reported in studies by Wang et al. [37], Shen et al. [38], and Zhai et al. [39], respectively. However, the effects of WAC and FEZ2 in HCC or on any other cancer have not been investigated.

In summary, among the prognostic genes we screened, the potential relationship among BIRC5, HMOX1, and VEGFA in HCC prognosis had been reported previously, while VPS35 might be a newly identified oncogene of HCC, and further, VPS26A and PRKCD were also found to have roles related to cancer. Thus, the available studies indicate that the prognostic genes selected are relatively reliable. As for WAC and FEZ2, for which a role in HCC or any other cancers has not been described, our research provides a rationale for further studies.

Autophagy has a dual role of promotion and suppression in cancer [40]. Based on the results of the present study, we speculate that autophagy is beneficial in the clinical outcomes of patients with HCC. Autophagy is a lysosome-dependent self-degradation process [41]. In the pathway enrichment results, our findings showed that the lysosomal pathway is inhibited in the high-risk group of patients (Figure 9), which indicates that in patients with poor prognosis, autophagy may be down-regulated. In addition, various studies have shown that autophagy could be induced to suppress HCC by inhibiting the PI3K/AKT/mTOR signaling pathway [42,43]. Downstream of mTOR signaling, the mTORC1 complex acts as an autophagy inhibitor by suppressing the expression of ULK1 [44]. From our results, we found that the mTOR pathway was activated in the high-risk group with poor prognosis (Figure 9), and the key autophagy gene ULK1 was significantly down-regulated (Table 2); therefore, indicating that autophagy activity is inhibited in high-risk HCC patients with poor prognosis. In summary, we speculate that autophagy is beneficial to the survival of HCC patients.

In our study, from the perspective of autophagy, we established an effective, stable, and reliable multigene predictive model for OS in HCC patients. This prognostic tool can be applied to newly diagnosed patients as follows: after measuring the expression value of eight prognostic genes for each patient on the same platform as the training set, the expression value is transformed by log2 (normalized RSEM count + 1), and then the risk score value is calculated. If the risk score is higher than the critical value, it is a high-risk patient, otherwise it is a low-risk patient. In addition, substituting the patient’s TNM stage and risk score values into the nomogram model can be used to predict the probability of the patient’s 1-, 3-, and 5- year OS. However, due to incomplete clinical data in the GEO dataset, our nomogram could not be externally verified in independent datasets. Therefore, future prospective studies are needed for the collection of new samples from multiple platforms and centers for clinical verification.

## Conclusions

Our results show that the autophagy-related risk model we established could effectively and independently predict the OS of HCC patients. The model has demonstrated robust cross-platform and cross-batch prediction capabilities. Patients’ risk scores can reflect the molecular status of HCC. Finally, our research provides new possibilities for determining prognoses and personalized therapeutics of HCC patients, and makes a significant contribution to research in preclinical medicine.

## Data Availability

The mRNA and corresponding phenotype data were obtained from UCSC Xena (https://tcga.xenahubs.net/download/TCGA.LIHC.sampleMap/HiSeqV2.gz; https://tcga.xenahubs.net/download/TCGA.LIHC.sampleMap/LIHC_clinicalMatrix) and GEO (GSE14520_GPL3921, GSE36376_GPL10558).

## Abbreviations

CI: confidence interval
GEO: Gene Expression Omnibus
HCC: hepatocellular carcinoma
HR: hazard ratio
OS: overall survival
TCGA: The Cancer Genome Atlas
